# Coccygeal Fracture-Dislocation in a Young Female: An Open Reduction Approach

**DOI:** 10.7759/cureus.94017

**Published:** 2025-10-07

**Authors:** Masashi Fujii, Yoshiaki Kimura, Hiroaki Kijima, Michio Hongo, Naohisa Miyakoshi

**Affiliations:** 1 Orthopedic Surgery, Akita City Hospital, Akita, JPN; 2 Orthopedic Surgery, Akita Hip Research Group (AHRG), Akita, JPN; 3 Orthopedic Surgery, Akita University Graduate School of Medicine, Akita, JPN; 4 Physical Therapy, Akita University Graduate School of Medicine, Akita, JPN; 5 Orthopedic Surgery, Akita Spine Group (ASG), Akita, JPN

**Keywords:** coccygeal alignment, coccyx, dislocation fracture, intrafocal pinning, open reduction

## Abstract

Reports of traumatic coccygeal dislocations or fracture-dislocations are rare, and there is no established consensus regarding their treatment. We report a case of traumatic anterior dislocating coccygeal fracture in a young female patient who initially underwent closed reduction, which was not feasible. A 17-year-old girl sustained an injury after falling backward down the stairs. Radiographs revealed anterior dislocation of the second coccygeal vertebra and distal segments, accompanied by small bone fragments, leading to the diagnosis of a traumatic coccygeal fracture-dislocation. An attempt at closed perianal reduction was made, but the dislocated second coccygeal vertebra was displaced proximally and overlapped with the first coccygeal vertebra. Furthermore, its distance from the anus was too large to prevent reduction. The approach was switched to open surgery. Reduction was achieved via a small incision using a technique similar to intrafocal pinning, and stability was achieved by applying axial compression. The pain resolved rapidly postoperatively. Although a mild alignment abnormality remained, the patient returned to sports activities eight weeks postoperatively without symptoms. Although correction of coccygeal alignment abnormalities is preferred, favorable reports exist regarding conservative treatment. Treatment selection should consider factors such as sex, age, and activity level.

## Introduction

In orthopedic outpatient clinics, many patients present with coccygeal pain after a backward fall. However, even if a fracture is present, approximately 90% experience pain improvement with conservative treatment [1], including oral analgesics, steroid injections, physical therapy, and pressure relief using a donut cushion [2]. However, residual alignment abnormalities or instability may result in persistent pain and limitations in daily activities such as walking, sitting, and defecation [3,4]. Some cases have reported unsuccessful conservative treatment, leading to coccygectomy more than three months post-injury [5-9]. The annual incidence rate of coccyx fracture was 119.75 per 100,000 individuals overall, with a rate of 33.44 per 100,000 in male patients and 86.30 per 100,000 in female patients [10]. Coccygeal fracture-dislocations are even rarer [11], and their diagnosis and treatment methods remain controversial. Restoring proper alignment after a dislocation is a standard goal, but methods vary, including both closed and open techniques, and there is no consensus on the subsequent fixation. We report the case of a young woman with an anterior dislocation with fracture of the coccyx following a fall, which could not be reduced by closed methods and required open surgical treatment.

## Case presentation

**Figure 1:**
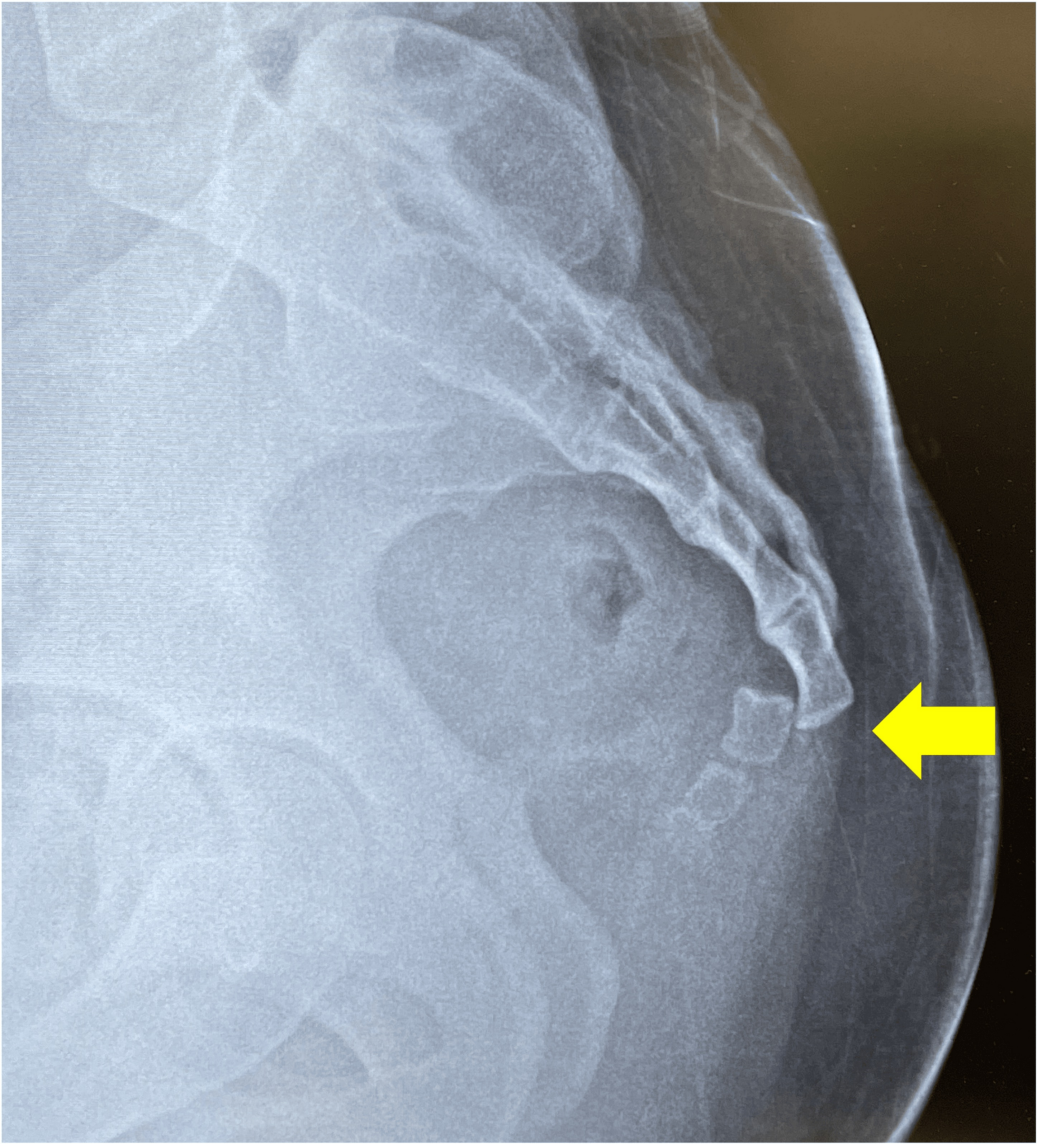
Imaging at the time of injury. Lateral radiograph showing anterior dislocation of the caudal vertebrae (arrow).

**Figure 2:**
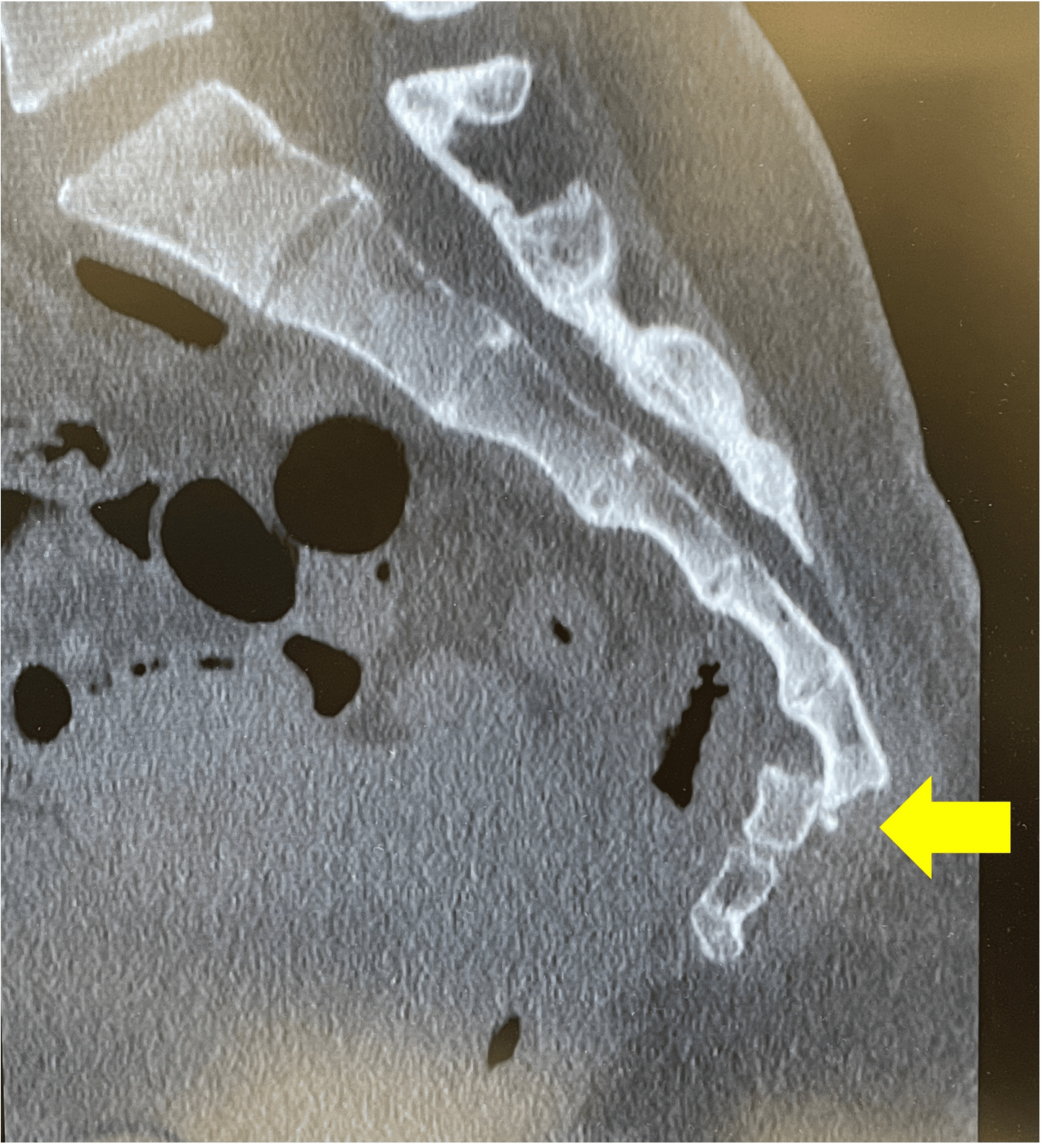
Imaging at the time of injury. Sagittal computed tomography (CT) slice showing a small bone fragment at the dislocation site (arrow).

The patient was a 17-year-old girl who was 164 cm tall and weighed 60 kg. She fell back from the second step of the staircase and forcefully struck her buttocks. She visited a local clinic where she was diagnosed with coccygeal dislocation and was referred to our department on the same day. She presented with spontaneous pain and tenderness in the coccyx and had difficulty moving while lying on the right side of the stretcher. Contusions and subcutaneous hemorrhage were noted at the injury site; however, there were no findings suggestive of neurological impairment, such as radiating pain to the lower limbs or sensory abnormalities. The patient had no relevant medical history. She was a member of her high school volleyball team. Plain lateral radiographs revealed a dislocation at the Coc1-2 level. The Coc2 vertebra and all vertebrae below were completely displaced ventrally, overlapping the Coc1 vertebra and exhibiting flexion deformity. Computed tomography (CT) scans showed small bone fragments suggestive of a fresh fracture and loss of intervertebral congruency (Figures 1, 2).

**Figure 3:**
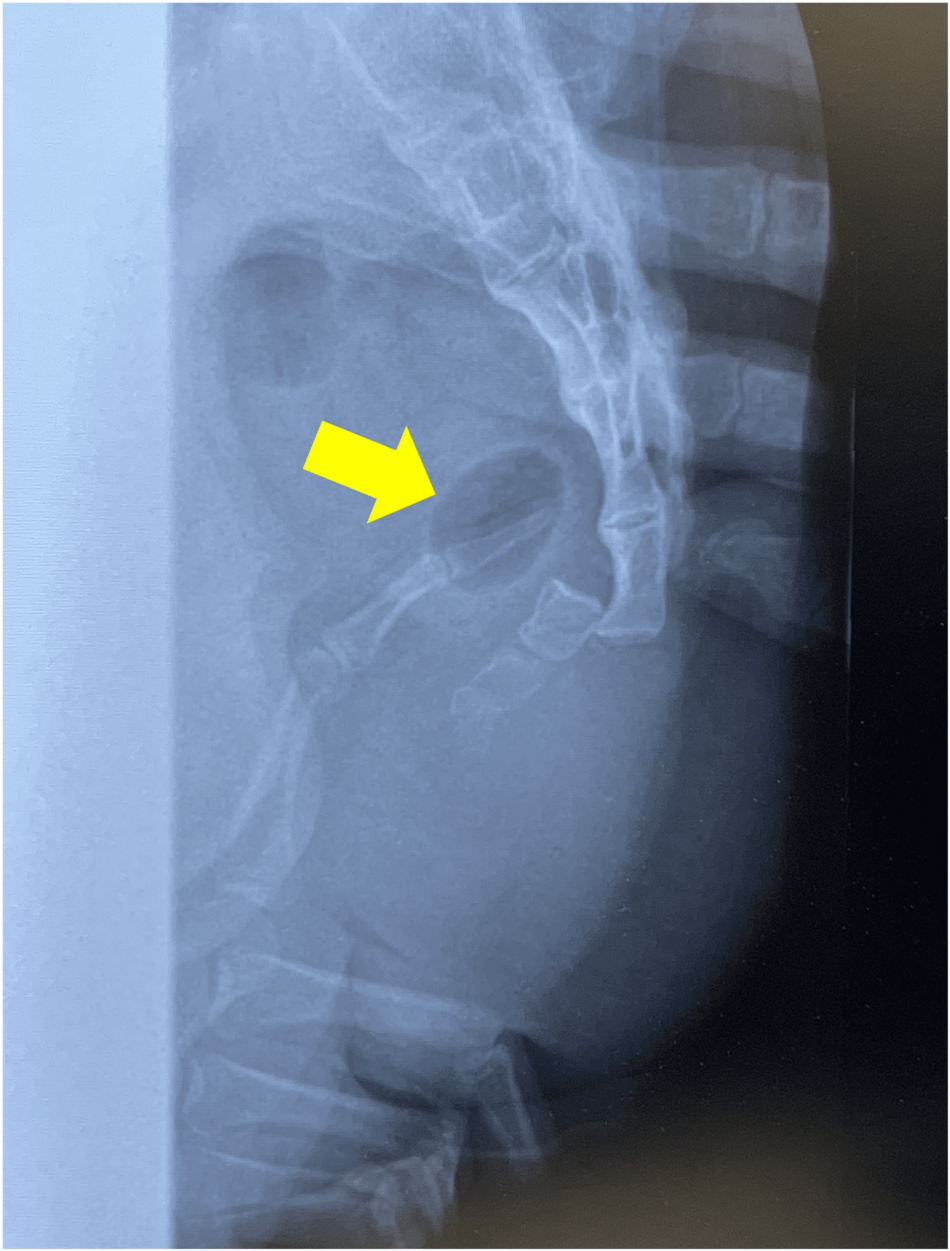
Closed reduction. Transanal reduction under X-ray guidance. The dislocation site (arrow) is at a depth barely reachable by the tip of the operator’s middle finger.

Based on these findings, a diagnosis of a traumatic anterior dislocation with fracture of the coccyx was made. After administering local anesthesia at the level of dislocation, manual reduction was attempted transanally under fluoroscopic guidance in accordance with previous reports [2]. However, the dislocation was interlocked, and the depth was such that the operator's middle finger could barely reach beyond the distal interphalangeal joint (Figure 3).

**Figure 4:**
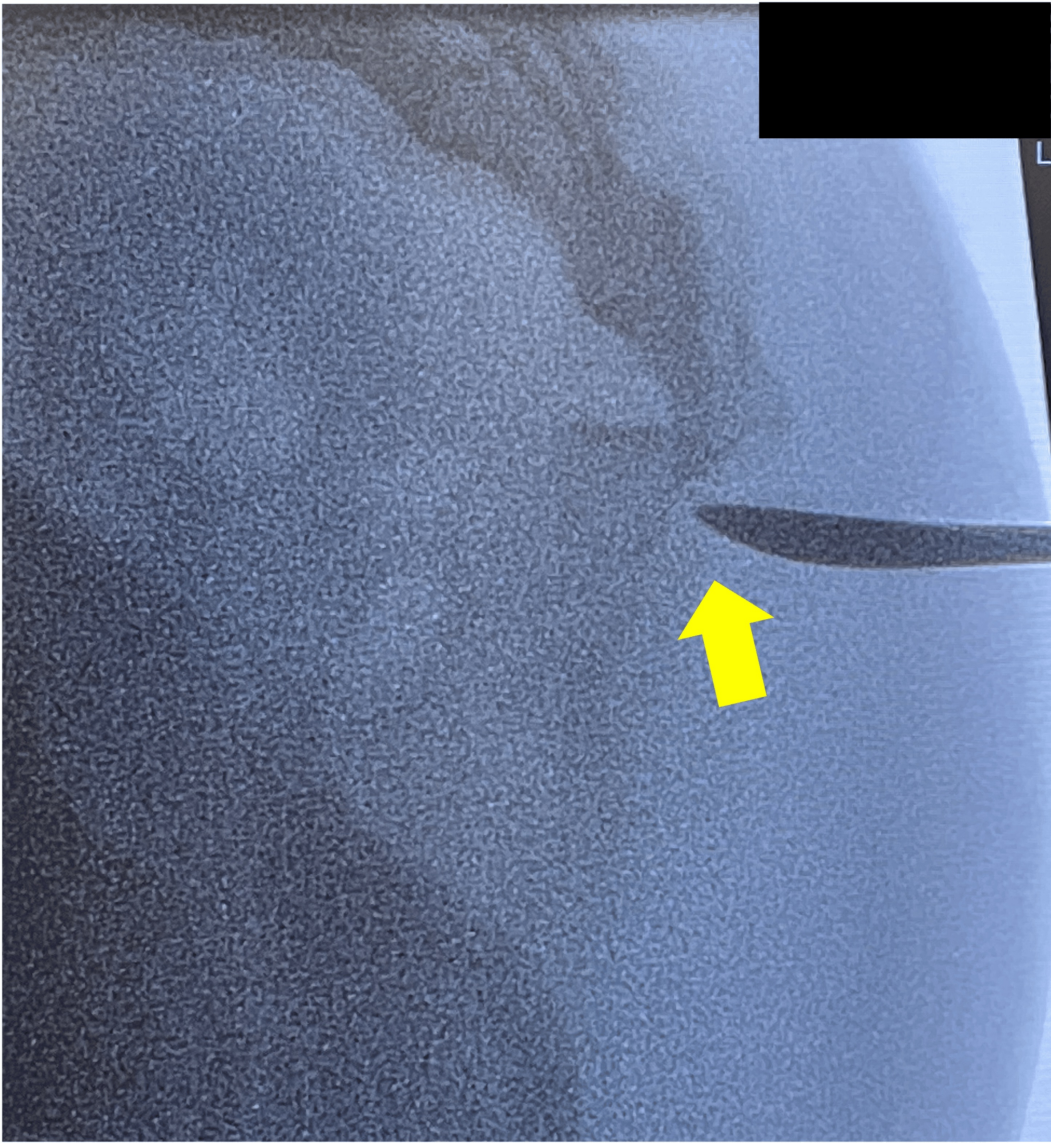
Open reduction. An elevator was inserted through the dislocation site (arrow).

**Figure 5:**
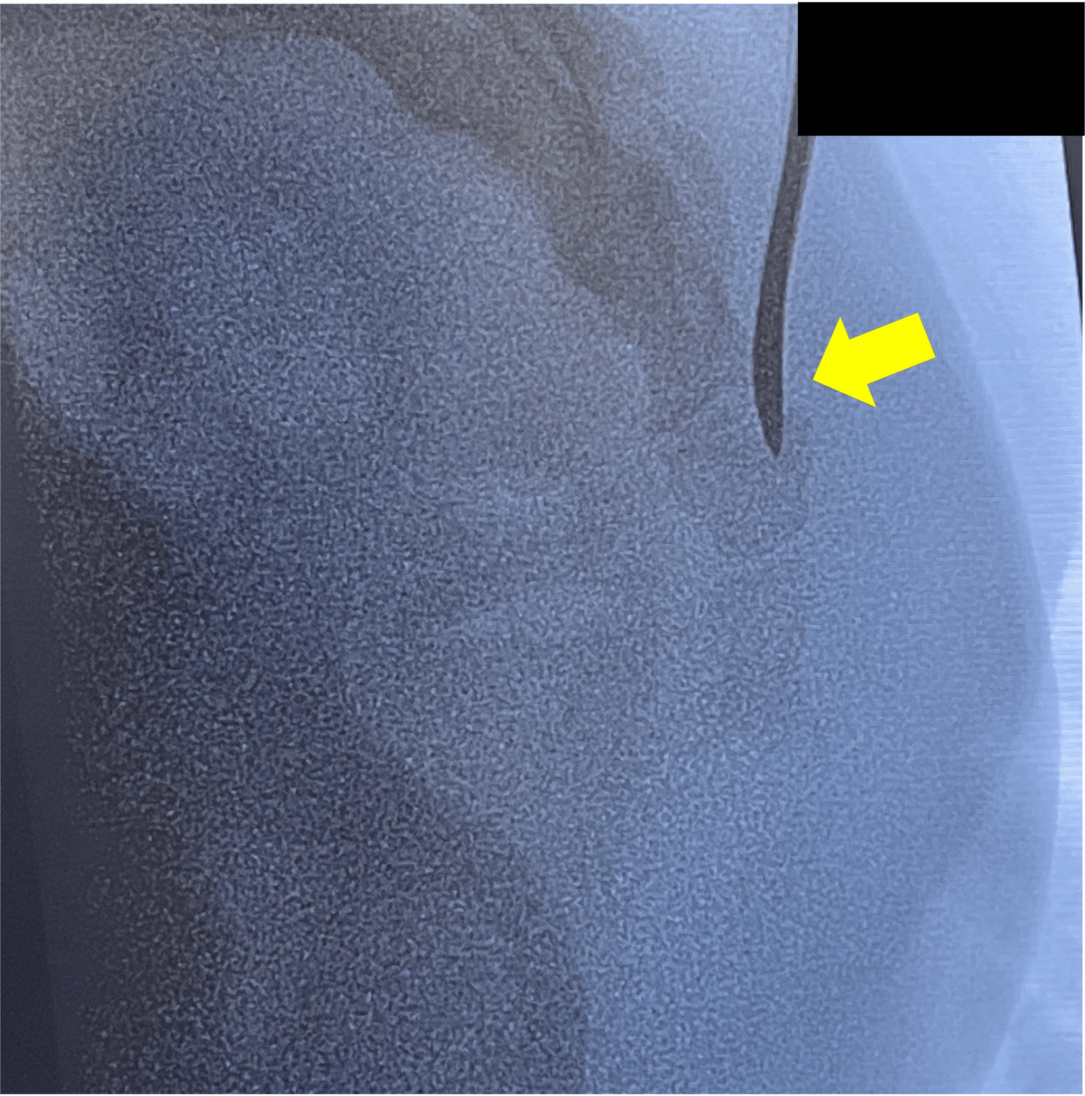
Open reduction. The reduction was performed according to the intrafocal pinning method (arrow).

**Figure 6:**
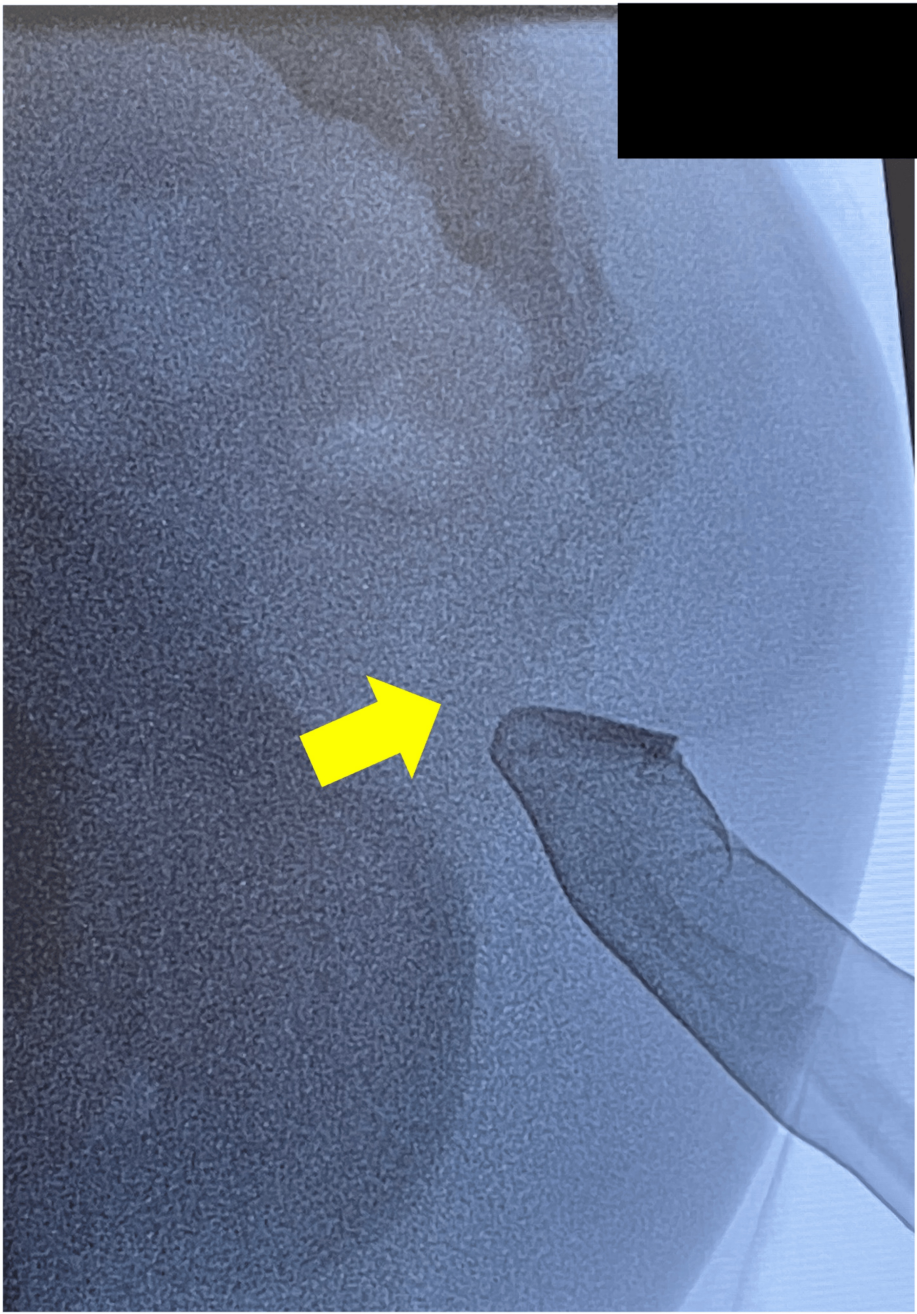
Open reduction. Stability was achieved by applying axial pressure distally to the dislocation site (arrow).

Sufficient external force could not be applied to the dislocation site, rendering reduction impossible. Therefore, the patient was transferred to the operating room for reduction under general anesthesia. The patient was placed in the right lateral decubitus position. A 1-cm longitudinal skin incision was made directly over the dislocation site for entry. After subcutaneously dissecting to the dislocation site, a periosteal elevator was inserted and levered on the distal coccyx to reduce it by pushing down and backward as previously reported [2]. Although slight instability persisted with residual separation between the dislocated vertebrae after reduction, applying axial compression by percutaneously pushing the distal fragment stabilized the joint. Therefore, internal fixation was not performed (Figures 4-6).

**Figure 7:**
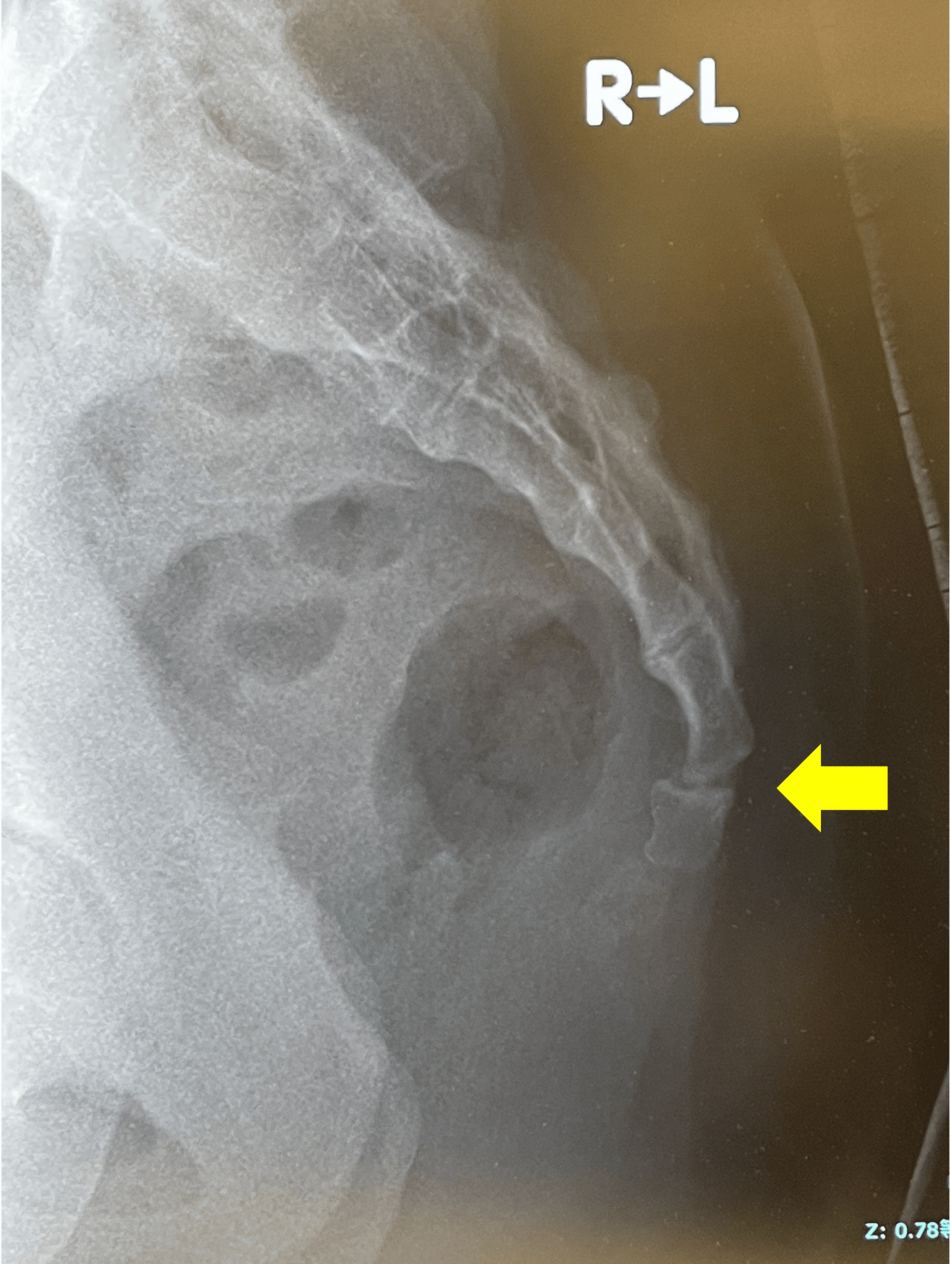
Examination at five weeks postoperatively. Mild alignment abnormality remains, but the fracture site has remodeled without redislocation (arrow).

Walking was permitted one day postoperatively, use of a Western-style toilet was allowed one week postoperatively, and sitting with a donut cushion was permitted four weeks postoperatively. At the five-week postoperative outpatient visit, radiographs showed slight subluxation without pain (Figure 7).

The patient returned to volleyball eight weeks postoperatively.

## Discussion

Coccygeal dislocation or fracture-dislocation is a rare injury with only limited reports in the literature, and its management remains controversial. According to a review by Panigrahi et al., conservative treatment was chosen in five of seven cases of sacrococcygeal joint dislocation, with generally favorable outcomes reported [12]. However, there have also been reports of residual symptoms after conservative treatment, leading to coccygectomy [5-9]. Therefore, it is necessary to consider a treatment plan tailored to each case.

Conservative treatments include the use of analgesics, soft cushions, and physical therapy. However, leaving the coccyx in a dislocated position carries the risk of chronic symptoms such as pain from direct irritation, discomfort, and defecation disorders. Therefore, achieving normal alignment is reasonable. Particularly in young women, consideration should be given to the potential effects on sexual intercourse and future childbirth [2].

**Figure 8:**
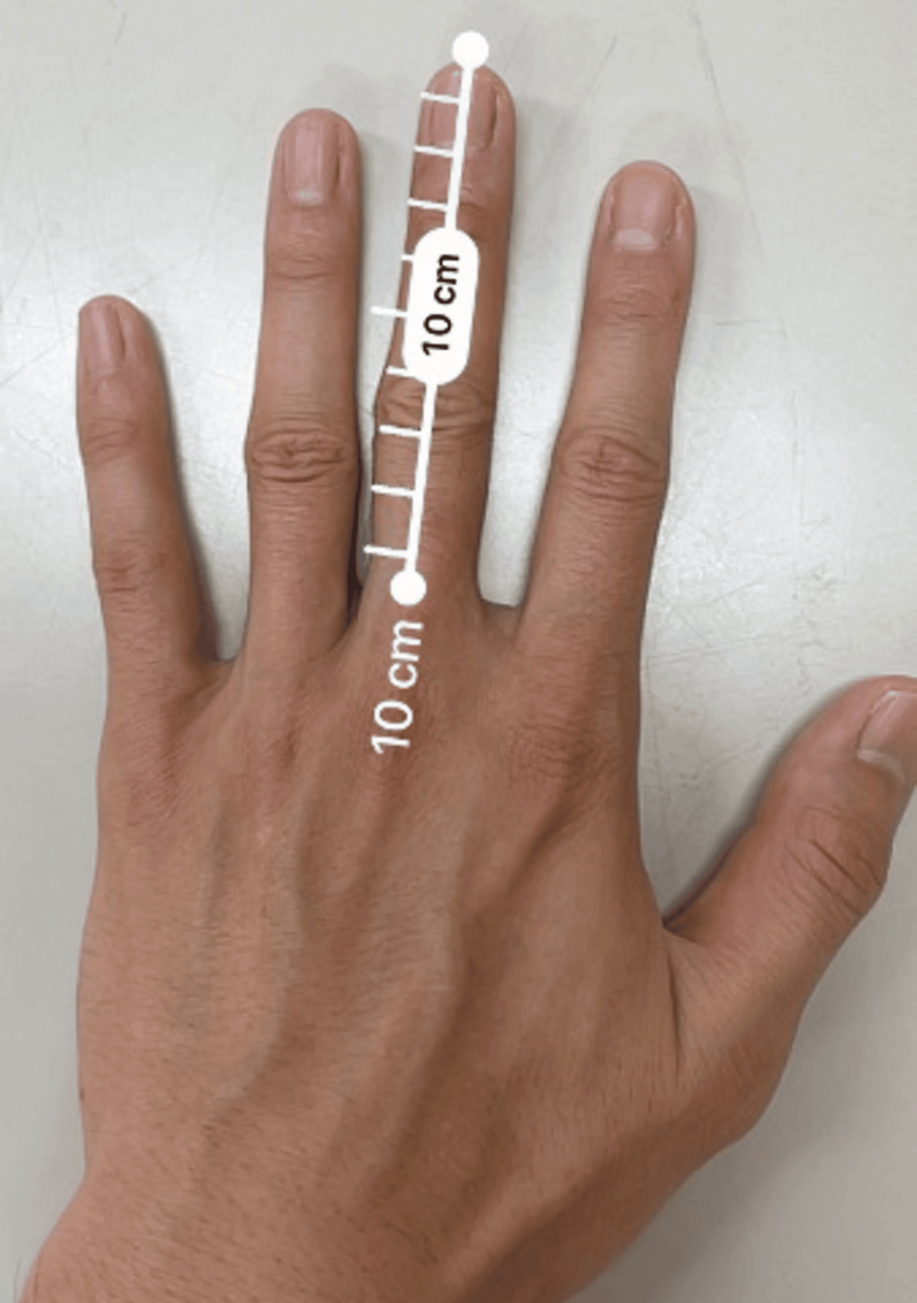
Operator's middle finger length.

Closed reduction via an image-guided transanal approach has also been reported. The technique involves grasping the distal aspect of the dislocation with the index or middle finger inserted transanally and the thumb outside the anus, then applying external force in the reduction direction [2,13]. Although it may be performed as a primary treatment after adequate pain relief and muscle relaxation, it requires significant force for reduction and carries the risk of rectal mucosal injury. Therefore, repeated procedures are not recommended [2]. Furthermore, even if reduction is achieved, additional measures should be considered if instability persists or redislocation occurs. Factors associated with successful reduction include young age, normal body mass index (BMI), and the absence of neurological symptoms. Although this case met these criteria, reduction was not achieved. The contributing factors likely included a high level of injury, interlocking at the dislocation site, and thickness of the gluteal soft tissues. Although the patient’s BMI was 22.3 kg/m², she had a consistent exercise routine and well-developed gluteal muscles. The operator was 186 cm tall with a middle finger length of 10 cm (Figure 8).

However, in this case, the gluteal soft tissue was thick, and the distal phalanx of the operator’s middle finger barely reached the dislocation site, preventing a sufficient reduction force from being transmitted. This was considered a factor in the failure to achieve reduction.

Open reduction is indicated for cases in which closed reduction fails or conservative treatment does not provide pain relief. Surgical treatment has been reported to achieve good alignment and favorable outcomes. Internal fixation with Kirschner wires is commonly chosen, although previous reports have described tension band fixation or fixation using sutures passed through the bone [2,14]. In the present case, the application of axial compression after open reduction stabilized the dislocation, allowing for a favorable outcome without additional fixation. After reduction, a stress test should be performed under fluoroscopy to carefully evaluate joint instability and the risk of redislocation. If instability persists after reduction, minimally invasive internal fixation should be performed.

Fracture-dislocation of the coccyx is rare, and there are significant individual variations in its original alignment. Particularly in females and those with a history of childbirth, the possibility that the dislocated position may have been originally well adapted must be considered. A detailed evaluation of local findings and the presence of traumatic changes on CT or magnetic resonance imaging at the time of injury is essential. As a case report, our findings are based on a single patient, and their generalizability is therefore limited. Treatment should be selected based on age, sex, and activity level.

## Conclusions

In this rare case of traumatic coccygeal fracture-dislocation in a young female, open reduction was performed considering the potential future effects on sexual intercourse and childbirth, yielding favorable results. Although reduction is important, treatment selection should balance the patient’s background and procedural invasiveness.
